# Cross-sectional study on the association between retinal microcirculation changes based on optical coherence tomography angiography and mild cognitive impairment in patients with type 2 diabetes

**DOI:** 10.3389/fmed.2025.1579562

**Published:** 2025-07-21

**Authors:** Wei Wang, Tao-Hong Zhang, Ling Jia

**Affiliations:** ^1^Department of Internal Medicine, Hefei Aier Eye Hospital Affiliated to Anhui Medical University, Hefei, Anhui, China; ^2^Department of Internal Medicine, Hefei Boe Hospital, Hefei, Anhui, China

**Keywords:** optical coherence tomography angiography, retinal microcirculation, type 2 diabetes mellitus, mild cognitive impairment, biomarker

## Abstract

**Objective:**

To investigate the association between retinal microcirculation changes, assessed using optical coherence tomography angiography (OCTA), and mild cognitive impairment (MCI) in patients with type 2 diabetes mellitus (T2DM), and to determine whether retinal microcirculation changes can serve as a potential biomarker for MCI in these patients.

**Methods:**

A total of 100 patients with T2DM who visited Hefei Aier Eye Hospital between April 2023 and December 2024 were selected. Retinal microcirculation indicators, including the perfusion density of the superficial capillary plexus (SCP), the perfusion density of the deep capillary plexus (DCP), the central foveal thickness (CMT) and the area of the foveal avascular zone (FAZ), were evaluated using OCTA. The cognitive function of the patients was assessed using the Montreal Cognitive Assessment. The association between retinal microcirculation indicators and MCI was explored using multivariate logistic regression analysis.

**Results:**

The perfusion density of SCP and DCP in all patients was lower than the normal value. Patients with CMT outside the normal range accounted for 73%, and those with FAZ outside the normal range accounted for 23%. No significant correlation was found between individual retinal microcirculation indicators and MCI. However, a significant association was observed when the four indicators were combined (*p* < 0.001), indicating that retinal microcirculation changes based on OCTA are significantly correlated with MCI in patients with T2DM.

**Conclusion:**

Retinal microcirculation changes based on OCTA are significantly associated with MCI in patients with T2DM, suggesting that retinal microcirculation indicators may serve as potential biomarkers for MCI in these patients.

## Introduction

Diabetes mellitus (DM) is a common metabolic disease characterized by elevated blood glucose levels. Type 2 diabetes mellitus (T2DM), which accounts for 90% of all diabetes cases, is primarily characterized by defects in insulin secretion from pancreatic *β*-cells and an inability of insulin-sensitive tissues to respond appropriately to insulin (1). According to 2021 statistics from the International Diabetes Federation, there were 537 million people with diabetes worldwide aged 20–79 years, and this number is projected to increase to 783 million by 2045. China has the highest prevalence of diabetes globally, with over 140 million patients, and the incidence is rising notably. It is estimated that by 2045, the number of patients with diabetes in China will reach 212 million (2, 3). Chronic, sustained hyperglycemia and significant blood glucose fluctuations can lead to various metabolic diseases, which, in turn, cause damage to vital organ functions and severely affect patients’ quality of life. Diabetes and its complications have become a major health threat to the Chinese population, imposing a heavy burden on individuals, families, and society.

In developed countries, microvascular complications caused by diabetes are the leading causes of blindness and end-stage renal disease (4). A report indicates that compared with healthy controls, patients with diabetes have reduced vascular density, particularly in the superficial and deep capillary plexuses (5). The retinal vascular network plays a crucial role in maintaining optimal retinal function. A comprehensive assessment of the structure and function of the retinal vascular system is essential for the diagnosis, therapeutic intervention and management of various retinal pathologies. Among diabetes-related complications, diabetic retinopathy (DR) is a common microvascular complication that can lead to acquired but preventable blindness. In industrialized countries, DR is the leading cause of blindness among working-age populations (6). The prevalence of DR varies by country, region and ethnicity, with higher rates in developed countries compared with developing countries (7). Studies have shown that the prevalence of DR in mainland China is 23%, with higher rates in rural areas compared with urban areas, and in the north compared with the south and east (8). Cognitive impairment (CI) is also a common complication of diabetes. This condition can be broadly categorized into three stages based on severity: preclinical cognitive decline, mild cognitive impairment (MCI) and dementia (9). The American Diabetes Association clearly stated in its 2021 guidelines that there is a significant correlation between diabetes and CI, and that the duration of diabetes is positively correlated with cognitive decline. It is emphasized that the issue of cognitive decline in patients with diabetes should be given high attention (10). Epidemiological studies have shown that patients with T2DM have a threefold higher risk of developing dementia compared with healthy individuals (11), with 20% of such patients over the age of 60 at risk of progressing to dementia (12). Previous studies have shown that the prevalence of MCI in patients with T2DM is approximately 20–30%, and the prevalence of dementia is around 17.3% (13, 14).

In recent years, an increasing number of studies have linked DR with CI (15). Both DR and CI share pathological mechanisms related to neurovascular factors, including neuroinflammation, neurodegeneration, gliosis and vascular degeneration (16). Chronic hyperglycemia and insulin resistance also play significant roles, leading to pathological mechanisms such as oxidative stress and the accumulation of advanced glycation end products. A study reported that microvascular dysfunction is one of the key mechanisms leading to cognitive decline in patients with diabetes (17). The microvascular system is involved in regulating many brain processes, and damage to it can induce stroke, depression and CI. Studies suggest a strong correlation between retinal pathology and CI, with notable similarities in the embryological origin, structure and physiological characteristics of retinal and cerebral microvasculature (18). Recent evidence indicates a link between retinal vascular abnormalities, CI and dementia, and that retinal neurovascular pathology may predict the progression of cognitive decline (17). However, although the pathological similarities are clear, the connection between retinal abnormalities and cognitive decline has not yet been fully established (19, 20).

Assessing retinal parameters through fundus examination to detect microvascular changes early may become an easier-to-accept method to reflect cerebral pathology and identify the risk of patients with T2DM developing CI and Alzheimer’s disease (AD) (21–23). The advent of optical coherence tomography angiography (OCTA) provides a method to quantify capillary blood flow and perfused vessel mapping. This method can automatically segment and visualize specific layers of the capillary plexus, displaying morphological characteristics such as peripapillary flow index, peripapillary retinal nerve fiber layer thickness and the number of neovessels on the disc in various fundus diseases, and these can be quantitatively analyzed. The method is non-invasive, convenient, easy to cooperate with and is considered an innovative tool for assessing the microvascular system in systemic diseases, and has been widely used in clinical practice (24, 25).

Optical coherence tomography angiography is a non-invasive imaging technique that is capable of providing high-resolution images of retinal microvessels. In recent years, with increased research interest in the association between DR and CI, OCTA has become an important tool for assessing changes in retinal microcirculation and its relationship with MCI. For example, Amato et al. (5) investigated the use of wide-field OCTA in DR and found that retinal microvessel density was significantly reduced in patients with diabetes, and this change may be related to cognitive dysfunction. In addition, Zhang and Liu (24) discussed the application of OCTA in ophthalmology, noting that this technology not only helps in the early detection of retinopathy but also serves as a window to assess the state of microvascular health throughout the body. Studies have shown that changes in retinal microvessels can reflect the state of brain microvessels due to their similarities in anatomical, embryological and pathological processes (16). Srikanth et al. (12) further noted that CI is an emerging complication of type 2 diabetes, and retinal microvascular changes may be one of the key predictors of this complication.

Given this, this study assesses retinal microcirculation changes in patients with T2DM using OCTA and explores their association with MCI, to reveal whether retinal microcirculation changes can serve as a potential biomarker for MCI in these patients. The study results will help to better understand the pathophysiological link between retinal microcirculation changes and CI, and provide a new perspective for the early identification and intervention of CI in patients with T2DM.

## Materials and methods

### Study population

A total of 100 patients with T2DM who were treated in Hefei Aier Eye Hospital between April 2023 and December 2024 were selected.

### Inclusion and exclusion criteria

The inclusion criteria were patients who met the diagnostic criteria for T2DM (26) and those with typical symptoms of diabetes (the three “polys” and one less) and random plasma glucose ≥ 11.1 mmol/L. For those without typical symptoms of diabetes, non-consecutive fasting venous plasma glucose ≥ 7.0 mmol/L on two occasions and/or 2-h blood glucose ≥ 11.1 mmol/L in an oral glucose tolerance test were required.

The exclusion criteria were as follows: (1) pregnant women; (2) patients with type 1 diabetes and those with a significant drop in hemoglobin levels due to bleeding, hemoglobinopathy and/or malignant tumors; (3) patients with a history of frequent hypoglycemia (≥2 major hypoglycemic events requiring assistance within the past 6 months, defined as blood glucose ≤ 3.9 mmol/L with impaired consciousness or other symptoms), recent diabetic ketoacidosis, hyperosmolar hyperglycemic syndrome, acute infection, trauma or other stress situations; (4) patients with severe dysfunction of vital organs such as heart, lung, liver and kidney, including acute myocardial infarction, chronic obstructive pulmonary disease (fev1 < 50% estimated), severe chronic hepatitis (prothrombin activity ≤ 40%, serum total bilirubin > 10 times normal), acute renal failure (serum creatinine rises ≥ 26.5 mmol/L within 48 h, or rises to more than 1.5 times the baseline value within 7 days) and chronic renal failure uremic phase; (5) patients with metabolic diseases that may affect cognitive function, such as hypertriglyceridemia (triglycerides [TG] ≥ 5.7 mmol/L) and hyperuricemia; (6) patients with endocrine diseases affecting cognitive function that are not controlled (thyroid-stimulating hormone abnormalities with obvious clinical symptoms), such as hypothyroidism and hyperthyroidism; (7) patients with neurological or psychiatric diseases, such as dementia, epilepsy, AD and mental disorders (e.g., depression, anxiety and schizophrenia) [tests are performed by taking a detailed medical history and performing a Montreal Cognitive Assessment (MoCA), and judging whether there is CI and its severity based on the score]; (8) patients with a history of hemorrhagic or ischemic stroke; (9) patients with a history of long-term alcohol abuse, carbon monoxide poisoning, drug addiction or use of drugs that affect cognitive function (including high-dose glucocorticoids, sedative-hypnotics, antipsychotics); (10) patients with other ophthalmic diseases that affect the fundus assessment of DR, such as severe cataracts, glaucoma, optic nerve atrophy, vitreous hemorrhage, central/branch retinal vein occlusion, retinal detachment, wet age-related macular degeneration (AMD) and previous fundus treatment (inclusion of patients with mild cataract or other ophthalmic diseases that do not affect OCTA examination was permitted); and (11) patients unable to complete all required assessment steps due to various reasons, such as communication difficulties or hearing and vision impairment.

### Data collection

Data on the patients’ demographic characteristics, clinical indicators, retinal microcirculation indicators and cognitive function assessment were collected.

In terms of demographic characteristics, the gender, age, educational level and duration of diabetes of the patients were recorded. At the same time, the patients’ blood lipid indicators, including total cholesterol (TC), TG, high-density lipoprotein cholesterol (HDL-C), low-density lipoprotein cholesterol (LDL-C), blood glucose indicators [including fasting plasma glucose (FPG) and glycated hemoglobin (HbA1c)] and other indicators [e.g., uric acid (UA)] were detected.

In this study, we used a Heidelberg Engineering’s SPECTRALIS® OCT2 device and its built-in OCTA module for all scans. Each scan covers a 3 × 3 mm area to ensure that the microvascular structure of each layer of the retina can be accurately captured. For image quality, we set strict thresholds, including a signal strength index ≥ 60 and no noticeable eye tracking artifacts or other distracting factors. Scans that did not meet these criteria were excluded. To assess inter-observer variability, two experienced readers independently reviewed a 10% random sample. The results showed that the score agreement between the two reached >90% (kappa coefficient >0.85), indicating good consistency.

Regarding retinal microcirculation indicators, OCTA was used to assess the perfusion density of the superficial capillary plexus (SCP) of the patients, the perfusion density of the deep capillary plexus (DCP), the central foveal thickness (CMT) and the area of the foveal avascular zone (FAZ).

The SCP perfusion density is usually expressed as a percentage (%), reflecting the blood flow perfusion of the superficial capillaries of the retina. In normal populations, the SCP perfusion density is typically between 90 and 100%. In patients with DR, the SCP perfusion density may be significantly reduced, generally below 90%, and the perfusion density will further decrease with the progression of the disease.

Similarly, the DCP perfusion density is usually expressed as a percentage (%), reflecting the blood flow perfusion of the deep capillaries of the retina. In normal populations, the DCP perfusion density is generally between 90 and 100%. In patients with DR, the DCP perfusion density may be significantly reduced, typically below 90%, and the perfusion density will further decrease with the progression of the disease.

The CMT is an important indicator for assessing the health of the macula, especially in the diagnosis and monitoring of macular diseases (e.g., macular edema, AMD). In normal populations, the CMT is usually between 180 and 260 μm. In macular edema, CMT significantly increases, generally exceeding 300 μm, and may even be higher. In AMD, CMT may vary depending on the type of lesion, with dry AMD typically having normal or slightly increased CMT, and wet AMD potentially undergoing a significant increase. In DR, CMT may significantly increase due to macular edema, generally exceeding 300 μm.

The FAZ is an avascular area in the center of the retinal macula, and changes in its area can reflect the health of the retinal vasculature. The area of FAZ is usually between 0.2 and 0.4 mm^2^. In DR, the area of FAZ may significantly increase, usually exceeding 0.4 mm^2^, and may even be higher. In retinal vein occlusion, the area of FAZ may significantly increase, typically exceeding 0.5 mm^2^. In macular diseases, such as macular edema and macular degeneration, the area of FAZ may increase.

In addition, the cognitive function status of the patients was assessed using the MoCA scale (27). During the baseline data collection phase, we recorded each participant’s highest educational level and divided it into three grades: primary school and below, secondary school and university and above. For the MoCA score, we added 1 point to the original score as the adjusted final score for participants with less than 12 years of education according to the internationally recommended adjustment rules. This adjustment was intended to reduce bias due to educational disparities. The total MoCA score is 30, with higher scores indicating better cognitive function. The common scoring criteria for MoCA are as follows:

- Normal cognitive function: score ≥ 26 points.
- MCI: primary school and below (6 years) ≤ 19 points, secondary school (12 years) ≤ 22 points, university ≤ 24 points.
- Dementia: score < 18 points.

### Calculation method of the combined OCTA indicators

In order to fully reflect the overall state of retinal microcirculation, we performed a comprehensive analysis of four OCTA parameters (SCP perfusion density, DCP perfusion density, CMT, and FAZ area): First, each parameter was normalized (z-score) to ensure that the data of different dimensions were comparable. Second, based on previous literature reports and actual data performance in this study, each parameter was given a certain weight (determined according to its relative importance in predicting MCI), and then the weighted mean was calculated as the combined OCTA score. The specific formula is as follows: Combined OCTA Score = w1 × SCP w2 × DCP w3 × CMT w4 × FAZ, where w1, w2, w3, and w4 represent the weights of each parameter. Finally, to ensure the validity and stability of the composite OCTA score, we perform an internal consistency test: by calculating Cronbach’s *α* coefficient, we evaluate the internal consistency between the four parameters. The results show α values > 0.7, indicating that these parameters have high internal consistency. 10% of the samples were randomly selected for repeated measurements and re-evaluated by two independent researchers. The Kappa coefficient > 0.85, indicating that the scoring results were reproducible.

### Statistical analysis

Statistical analysis was performed using SPSS version 26.0 (IBM Corp., Armonk, New York). Normality tests were conducted for quantitative data, with those meeting the normal distribution expressed as mean ± standard deviation and those that did not described using the median (P25–P75). Categorical data were presented as frequency (%). Comparisons of quantitative data between two groups that met the normal distribution were made using the independent samples t-test; comparisons of quantitative data that did not meet the normal distribution were made using the non-parametric rank-sum test; and comparisons of categorical data between two groups were made using the chi-square test. Correlation analysis was performed using univariate linear regression analysis and logistic regression analysis. To identify independent predictors of MCI, multivariate logistic regression analysis was conducted. All covariates were entered simultaneously using the enter method to adjust for potential confounding factors. Odds ratios (ORs) with 95% confidence intervals (CIs) were calculated. A *p*-value of <0.05 was considered statistically significant.

## Results

The baseline characteristics of the study participants are shown in Table 1. In this study, a total of 100 patients were included, of whom 58 were men (58%) and 42 were women (42%). The average educational level of the patients was 12.3 ± 2.7 years. The average age of the patients was 61.73 ± 9.61 years. The average duration of T2DM was 5.04 ± 4.57 years.

**Table 1:** Patient baseline characteristics, retinal microcirculation indicators, and MoCA scores.

Risk factors	Total	Male (58/100, 58%)	Female (42/100, 42%)
Age (years)	61.73 ± 9.61	60.2 ± 10.1	60.8 ± 11.2
Educational level (years)	12.3 ± 2.7	11.8 ± 3.2	13.3 ± 1.7
2DM (years)	5.04 ± 4.57	3.6 ± 4.8	3 ± 4.9
TC (mmol/L)	4.53 ± 1.18	4.7 ± 1.2	4.4 ± 1
TG (mmol/L)	1.57 ± 1.14	1.6 ± 0.9	1.7 ± 1
HDL-C (mmol/L)	1.29 ± 0.41	1.4 ± 0.5	1.4 ± 0.3
LCL-C (mmol/L)	2.34 ± 0.86	2.3 ± 0.8	2.1 ± 0.7
FPG (mmol/L)	6.84 ± 2.11	7 ± 2.3	6.3 ± 1.6
UA (μmol/L)	291.44 ± 114.27	320 ± 87.6	250.6 ± 82.2
HbAlc (%)	6.64 ± 1.83	6.8 ± 1.5	6.7 ± 1.6
The perfusion density of SCP (%)	46.14 ± 8.11	45.4 ± 5.3	47 ± 5.3
The perfusion density of DCP (%)	45.67 ± 7.12	45.6 ± 6.2	45.4 ± 6.8
CMT (μm)	304.17 ± 85.45	296.9 ± 70.2	280.7 ± 60.9
FAZ (mm^2^)	0.33 ± 0.18	0.3 ± 0.2	0.4 ± 0.2
MoCA scores	23.17 ± 1.61	22.7 ± 1.6	23.4 ± 1.5
MCI (%)	21.0%	22.4%	19.0%

MoCA, Montreal Cognitive Assessment; TG, triglycerides; TC, total cholesterol; HDL-C, high-density lipoprotein cholesterol; LDL-C, low-density lipoprotein cholesterol; FPG, fasting plasma glucose; UA, uric acid; HbA1c, glycated hemoglobin; SCP, superficial capillary; DCP, deep capillary plexus; CMT, central foveal thickness; FAZ, foveal avascular zone; MCI, mild cognitive impairment.

Regarding lipid profile indicators, the average TC was 4.53 ± 1.18 mmol/L (Table 1), and 21 patients (15 men and 6 women) had abnormal TC values (Table 2). The average TG was 1.57 ± 1.14 mmol/L, and 37 patients (20 men and 17 women) had abnormal values. The average HDL-C was 1.29 ± 0.41 mmol/L, and 7 patients (6 men and 1 woman) had abnormal values. The average LDL-C was 2.34 ± 0.86 mmol/L, and 7 patients (5 men and 2 women) had abnormal values.

**Table 2:** Number of patients with each indicator outside the normal range.

Risk factors	Standard level	Total	Male (58/100, 58%)	Female (42/100, 42%)
TC (mmol/L)	<5.2	21	15	6
TG (mmol/L)	<1.7	37	20	17
HDL-C (mmol/L)	≥1	7	6	1
LCL-C (mmol/L)	<3.4	7	5	2
FPG (mmol/L)	3.9–6.1	62	39	23
UA (μmol/L)	204–416.4	12	10	2
HbAlc (%)	<5.7	82	49	33
The perfusion density of SCP (%)	90–100%	100	58	42
The perfusion density of DCP (%)	90–100%	100	58	42
CMT (μm)	180–260	73	45	28
FAZ (mm^2^)	0.2–0.4	23	14	9
MoCA scores	≥26	100	58	42

MoCA, Montreal Cognitive Assessment; TG, triglycerides; TC, total cholesterol; HDL-C, high-density lipoprotein cholesterol; LDL-C, low-density lipoprotein cholesterol; FPG, fasting plasma glucose; UA, uric acid; HbA1c, glycated hemoglobin; SCP, superficial capillary; DCP, deep capillary plexus; CMT, central foveal thickness; FAZ, foveal avascular zone.

In terms of other indicators, the average FPG was 6.84 ± 2.11 mmol/L, and 62 patients (39 men and 23 women) had abnormal values. The average HbA1c was 6.64 ± 1.83%, and 82 patients (49 men and 33 women) had abnormal values. The average UA was 291.44 ± 114.27 μmol/L, and 12 patients (10 men and 2 women) had abnormal values. The average score on the MoCA was 23.17 ± 1.61, with an average of 22.7 ± 1.6 for men and 23.4 ± 1.5 for women. All patients scored between 18 and 25, indicating MCI (Table 1).

To better understand the retinal microcirculation of the patients, we used OCTA to assess the perfusion density of the SCP, the perfusion density of the DCP, the CMT and the FAZ (Table 1). The average SCP perfusion density was 46.14 ± 8.11%, the average DCP perfusion density was 45.67 ± 7.12%, the average CMT was 304.17 ± 85.45 μm and the average FAZ was 0.33 ± 0.18 mm^2^. As Table 2 shows, all patients had SCP and DCP perfusion densities below the normal range. There were 73 patients with CMT outside the normal range (180–260 μm), including 45 men and 28 women. There were 23 patients with FAZ outside the normal range (0.2–0.4 mm^2^), including 14 men and 9 women. The above results indicate that all patients had varying degrees of retinal microcirculation changes.

To investigate the correlation between changes in retinal microcirculation indicators detected by OCTA and MCI in patients with T2DM, we performed correlation analysis between SCP perfusion density, DCP perfusion density, CMT and FAZ and MoCA scores (Table 3). The results showed that none of the four OCTA indicators were significantly correlated with CI. Subsequently, we conducted a multivariable logistic regression analysis to further investigate the impact of the OCTA indicators of these four combinations on the MCI scores (Table 4). At the same time, age, gender, educational level, diabetes course and various biochemical indicators were included in the regression model to eliminate interference factors. The results showed that the OCTA indicators of the combinations were significantly associated with the MoCA scores (*p* < 0.001), suggesting that the overall state of retinal microcirculation may more accurately reflect the risk of MCI in patients with T2DM than any single indicator.

**Table 3:** Analysis of the correlation between retinal microcirculation indicators and MoCA scores.

Item	Pearson correlation coefficient r with MoCA scores (95% CI)	*p* value
The perfusion density of SCP (%)	−0.007 (−0.203 to 0.190)	0.945
The perfusion density of DCP (%)	0.075 (−0.123 to 0.267)	0.459
CMT (μm)	0.006 (−0.191 to 0.202)	0.956
FAZ (mm^2^)	−0.086 (−0.278 to 0.112)	0.395

SCP, superficial capillary; DCP, deep capillary plexus; CMT, central foveal thickness; FAZ, foveal avascular zone.

**Table 4:** Logistic multivariate analysis of independent predictors for MCI.

Item	OR (95% CI)	*p* value
The perfusion density of SCP (%)	1.056 (0.923–1.208)	<0.001
The perfusion density of DCP (%)	0.985 (0.882–1.099)	<0.001
CMT (μm)	0.999 (0.992–1.007)	<0.001
FAZ (mm^2^)	1.020 (0.937–1.110)	<0.001

SCP, superficial capillary; DCP, deep capillary plexus; CMT, central foveal thickness; FAZ, foveal avascular zone; MCI, mild cognitive impairment.

## Discussion

This study investigates the association between retinal microcirculation changes, assessed using OCTA, and MCI in patients with T2DM. The study results show that the retinal microcirculation indicators of patients with T2DM are significantly abnormal, and that these indicators are significantly associated with MCI. This finding provides a new perspective for understanding the potential link between retinal microcirculation changes and MCI, and suggests that retinal microcirculation changes may serve as a potential biomarker for CI in patients with T2DM.

In this study, the SCP and DCP of all participants were below the normal value. There were 73 patients (73%) with CMT outside the normal range, and 23 (23%) with FAZ outside the normal range, indicating that retinal microcirculation abnormalities are common in patients with T2DM. This is similar to the findings of previous studies. Yao et al. (28) conducted a cross-sectional study based on OCTA involving 860 participants (including 449 patients with diabetes and 411 without diabetes) and found that the retinal microvascular density (including perfusion density and vascular density) of patients with diabetes was significantly reduced and closely related to elevated fasting blood glucose levels, indicating that hyperglycemia may be an important factor causing retinal microvascular damage.

The present study’s results further show that there is a significant association between retinal microcirculation indicators and MCI, which is consistent with the link between DR and CI found in previous studies. As a highly vascularized tissue, the retina is susceptible to microvascular damage, and retinal imaging provides a non-invasive method for detecting subtle changes. The microvascular status in the retinal vasculature often reflects systemic or cardiovascular conditions due to their similar vascular scale and pathology (28). Since the retina is a brain-derived tissue that develops individually, vascular changes occurring in other parts of the body are logically considered to occur similarly in the brain (23). According to this theory, the assessment of retinal microvasculature will reflect subtle brain lesions because the retina and the brain have similar anatomical, embryological and physiological characteristics, as well as similar pathological processes and aging mechanisms (19). The blood–brain barrier and the blood–retina barrier regulate the supply of oxygen and glucose to brain tissue and the retina, preventing exposure of nerve cells and the central nervous system to pathogens and toxic substances, thereby providing protection for the central nervous system and the retinal microenvironment. Retinal gliosis, neurodegeneration and microvascular dysfunction are interdependent in the development of DR (22), meaning retinal microcirculation changes with both microcirculation and neurodegenerative changes may predict similar changes in the brain’s microvasculature, thereby affecting cognitive function.

Retinal microcirculation affects cognitive performance in patients with diabetes through a variety of pathways. The retinal vasculature is the window of the small blood vessel system throughout the body, and the changes in its microcirculation can reflect the state of the small blood vessels in the brain. Chronic hyperglycemic states in patients with DM may lead to endothelial cell dysfunction in the retina and small blood vessels in the brain, increased inflammation and increased oxidative stress, which may combine to impair vascular structure and function and thus affect cognitive function (12, 17, 22). Specifically, long-term hyperglycemia can lead to increased retinal microvascular permeability, hemodynamic changes and even neovascularization, which can likewise occur in brain microvessels, leading to neuronal metabolic disorders and dysfunction. In addition, diabetes-related neurodegeneration may also affect cognitive function through a variety of pathways, including insulin resistance, mitochondrial dysfunction and tau protein abnormalities.

Several studies support the use of OCTA to explore the link between retinal microvascular changes and cognitive dysfunction. For example, a systematic review and meta-analysis conducted by Wu et al. (18) showed a significant association between DR and CI, suggesting that retinal microvascular abnormalities may be an important marker of cognitive decline. In addition, the cumulative evidence in observational studies by Chai et al. (23) suggests an association between DR and structural brain abnormalities and CI, which provides a theoretical basis for retinal microvascular changes as an early screening indicator for CI. Recent studies have further highlighted the potential of retinal microvascular states as a “window” into the health of the brain’s microvessels. For example, Simó-Servat et al. (21) found that in patients with AD, abnormalities in retinal microcirculation detected by OCTA (e.g., decreased capillary density) were strongly associated with CI, suggesting that retinal microangiopathy may reflect similar changes in brain microvasculature. Similarly, Luis et al.’s study showed a significant association between retinal microcirculation impairment and cognitive decline in older adults with diabetes (27). These studies suggest that changes in retinal microvessels not only help to understand the relationship between DR and CI but also provide a new perspective for early screening and intervention using OCTA technology.

Interestingly, no significant correlation was found between individual retinal microcirculation indicators and MCI, but a significant association was observed when the four indicators were combined. This phenomenon may be related to the complexity and multidimensional nature of retinal microcirculation changes. Each retinal microcirculation indicator reflects different aspects of the retinal microcirculation system. For example, SCP perfusion density mainly reflects the blood flow perfusion of the superficial capillaries of the retina, whereas DCP perfusion density reflects the perfusion of the deep capillaries; CMT mainly reflects structural changes in the macular area, whereas FAZ area reflects the size of the avascular area in the macular center (29). These indicators reflect the overall state of retinal microcirculation from different perspectives. When analyzed individually, each indicator may only provide limited information and fail to comprehensively reflect the complexity of retinal microcirculation. However, when these four indicators are combined, they can complement each other and more comprehensively reflect the overall changes in retinal microcirculation, thereby establishing a significant association with CI. In addition, retinal microcirculation changes may be related to multiple pathological mechanisms that collectively contribute to the occurrence and development of CI. For example, oxidative stress caused by chronic hyperglycemia, accumulation of advanced glycation end products and microvascular dysfunction can all lead to damage to the retinal and brain microvasculature (29). These injuries may manifest as different degrees of changes in different retinal microcirculation indicators. When analyzed individually, each indicator may only reflect part of the pathological process, meaning the association with CI is not significant. However, when multiple indicators are considered together, the cumulative effect of the various pathological processes reflected by these indicators may be more significantly associated with CI. This result suggests that when evaluating CI in T2DM, multiple retinal microcirculation indicators should be considered to more comprehensively reflect the overall state of retinal microcirculation and its potential link with CI.

Although this study revealed a significant association between retinal microcirculation changes and MCI, there are still some limitations. First, this is a cross-sectional study and cannot determine a causal relationship. Future longitudinal studies are needed to further explore the causal relationship between retinal microcirculation changes and CI and their trends over time. Second, the sample size is relatively small, and the study population mainly come from a single area, which may limit the generalizability of the study results. Future studies should expand the sample size and cover a broader population to verify the findings of this study. In addition, this study did not conduct a detailed assessment of other factors that may affect cognitive function (e.g., lifestyle), which may have some impact on the study results. Furthermore, there was no control group (e.g., healthy individuals or patients without diabetes) in this study, which makes it difficult to determine whether OCTA changes are specifically associated with MCI or simply a general effect of diabetes or the aging process. Future studies should consider including a control group to better assess these differences. Finally, detailed analysis of different subgroups (e.g., stratification by duration of diabetes, retinopathy severity) was lacking. This hierarchical analysis could help reveal important associations and provide a deeper understanding. Specifically, the DR itself may affect the OCTA metrics independently, and not adjusting the DR status may confound the results. Therefore, DR should be considered as a confounding factor in subsequent studies.

## Conclusion

This study assessed the retinal microcirculation changes in patients with T2DM using OCTA and revealed a significant association with MCI. This finding provides new evidence for understanding the pathophysiological link between DR and MCI and suggests that retinal microcirculation indicators may serve as potential biomarkers for MCI in patients with T2DM.

## Data Availability

The original contributions presented in the study are included in the article/supplementary material, further inquiries can be directed to the corresponding author.
